# Work-loop contractions reveal that the afterload-dependent time course of cardiac Ca^2+^ transients is modulated by preload

**DOI:** 10.1152/japplphysiol.00137.2022

**Published:** 2022-06-30

**Authors:** Jarrah M. Dowrick, Kenneth Tran, Amy S. Garrett, Alex J. Anderson, Poul M. F. Nielsen, Andrew J. Taberner, June-Chiew Han

**Affiliations:** ^1^Auckland Bioengineering Institute, University of Auckland, Auckland, New Zealand; ^2^Department of Engineering Science, University of Auckland, Auckland, New Zealand

**Keywords:** afterload, Ca^2+^ handling, Ca^2+^ transient, preload, work-loop

## Abstract

Preload and afterload dictate the dynamics of the cyclical work-loop contraction that the heart undergoes in vivo. Cellular Ca^2+^ dynamics drive contraction, but the effects of afterload alone on the Ca^2+^ transient are inconclusive. To our knowledge, no study has investigated whether the putative afterload dependence of the Ca^2+^ transient is preload dependent. This study is designed to provide the first insight into the Ca^2+^ handling of cardiac trabeculae undergoing work-loop contractions, with the aim to examine whether the conflicting afterload dependency of the Ca^2+^ transient can be accounted for by considering preload under isometric and physiological work-loop contractions. Thus, we subjected ex vivo rat right-ventricular trabeculae, loaded with the fluorescent dye Fura-2, to work-loop contractions over a wide range of afterloads at two preloads while measuring stress, length changes, and Ca^2+^ transients. Work-loop control was implemented with a real-time Windkessel model to mimic the contraction patterns of the heart in vivo. We extracted a range of metrics from the measured steady-state twitch stress and Ca^2+^ transients, including the amplitudes, time courses, rates of rise, and integrals. Results show that parameters of stress were afterload and preload dependent. In contrast, the parameters associated with Ca^2+^ transients displayed a mixed dependence on afterload and preload. Most notably, its time course was afterload dependent, an effect augmented at the greater preload. This study reveals that the afterload dependence of cardiac Ca^2+^ transients is modulated by preload, which brings the study of Ca^2+^ transients during isometric contractions into question when aiming to understand physiological Ca^2+^ handling.

**NEW & NOTEWORTHY** This study is the first examination of Ca^2+^ handling in trabeculae undergoing work-loop contractions. These data reveal that reducing preload diminishes the influence of afterload on the decay phase of the cardiac Ca^2+^ transient. This is significant as it reconciles inconsistencies in the literature regarding the influence of external loads on cardiac Ca^2+^ handling. Furthermore, these findings highlight discrepancies between Ca^2+^ handling during isometric and work-loop contractions in cardiac trabeculae operating at their optimal length.

## Graphical abstract

**Figure:**
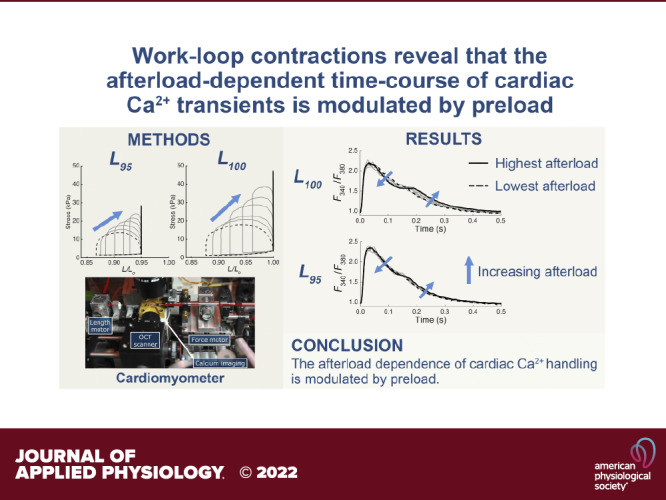

## INTRODUCTION

The heart in vivo undergoes a cyclical work-loop contraction pattern where the venous pressures upstream (preload) and arterial pressures downstream (afterload) dictate the pressure development and, therefore, the shortening and lengthening dynamics of ventricular muscle fibers. Over the past century, it has become apparent that cardiac stress-length mechanics are contraction mode dependent. For a given length, the isometric stress production is greater than the end-systolic stress produced at the end of the shortening phase in a work-loop, and this discrepancy is augmented at lower afterloads (1) but eliminated at reduced preloads (2).

Ca^2+^ transients, the primary driver of cardiac contraction, are commonly studied within isometric contractions, where an external load prevents isolated muscle fibers from shortening. No study has measured Ca^2+^ transients in cardiac trabeculae undergoing work-loop contractions. A handful of investigations, in both multicellular (3–5) and single cellular (6, 7) preparations, have examined the influence of afterloaded shortening on the Ca^2+^ transient but using isotonic contraction modes. The general finding was that, at low afterloads, the twitch duration reduced, whereas the Ca^2+^ transient amplitude did not change and the transient duration extended.

Ca^2+^ transients have been measured during work-loop contractions in whole hearts (8), but the study was limited to a single afterload at the mid-range of isovolumic pressure. Under these conditions, Ca^2+^ transients demonstrated no contraction-mode dependency. That is, the Ca^2+^ transient under isovolumic and work-loop conditions was indistinguishable. This sets a precedent for an equivalency of work-loop and isometric Ca^2+^ handling that may be unfounded over a greater range of afterloads.

Mode-dependency of Ca^2+^ handling has not been resolved in silico. Mathematical models of isotonic shortening have predicted that the Ca^2+^ transient duration either increases (9) or decreases (10) at reduced afterloads. Modeling studies focused on work-loop contractions, where the muscle is allowed to fully relax before restretch occurs, predict a morphological change to the decay of the Ca^2+^ transient but little to no change to the duration with changing afterload (11, 12).

The preload dependence of Ca^2+^ handling in cardiac muscle has been studied in isometrically contracting trabeculae (13–15) and auxotonically contracting myocytes (16), but it has not yet been examined in isolated cardiac muscle undergoing work-loop contractions. From a mechanistic standpoint, preload has a major role in modulating the ability of cardiac muscle to relax under afterloaded isotonic contractions in trabeculae (17) and myocytes (10, 18), as well as on the mechano-Ca^2+^ feedback as recently shown in silico (19). Hence, it is possible that preload modulates the afterload-dependent Ca^2+^ handling observed in the literature.

The aim of conducting the experiments in this study is to gain an understanding of the Ca^2+^ handling of cardiac trabeculae performing work-loops. The experiments involved subjecting isolated ventricular trabeculae to work-loops at two preloads and a range of afterloads in the custom-built cardiomyometer. Preload was set by prestretching the muscle to 95% or 100% optimal length. Steady-state twitch stress and Ca^2+^ transient data were measured and analyzed to assess their responses to these loading conditions. By collecting these data, we provide the first insight into the influence of preload on Ca^2+^ handling during work-loop contractions over a wide range of afterloads, thereby reconciling a literature inconsistency.

## MATERIALS AND METHODS

### Ethical Approval

The Animal Ethics Committee of The University of Auckland approved protocols for animal handling and euthanasia (AEC No. 2722).

### Work-Loop Control

All experiments were conducted on isolated ventricular trabeculae from male Wistar rats in the cardiomyometer (20, 21). In this device, trabeculae developed force-length work loops by contracting against a mechanical admittance computed in real time in a field-programmable gate array (FPGA) and imposed by a linear actuator based on a voice coil motor (22). The mechanical admittance (velocity per unit force) experienced by the muscle was scaled from the instantaneous solution of a three-element “Windkessel” model of the vasculature impedance (*Z_wk_*) as follows. Briefly, the force generated by the muscle upon stimulation was scaled in the FPGA to an equivalent ventricular pressure using measured muscle dimensions and applying Laplace’s law (23). This scaling technique approximates the ventricle as a sphere due to the timing and hardware constraints of the FPGA used. Although a different geometrical model would likely change the morphology of the stress-length loops, the model used was sufficient to test the hypothesis that the Ca^2+^ transient is load-dependent, without introducing bias.

The muscle contracted isometrically until the pressure exceeded *Pa*(s), the Laplace domain expression of the arterial pressure stored in the vascular network. As in the heart, ejection/muscle shortening could only occur when ventricular pressure exceeded that of the arterial network. Once this condition was met, the flow rate of blood out of the ventricle was computed from

(*1*) Qs=PasZWks

where *Z_Wk_*(s) is the Laplace-domain transfer function of a Windkessel model of the vasculature defined as

(*2*) $$Z_{Wk}\left(s\right)=\frac{\left(Z_{c}R_{p}C\right)s+\left(Z_{c}+R_{p}\right)}{\left(R_{p}C\right)s+1}$$

*Z_c_* is the characteristic impedance, *R_p_* is the peripheral resistance, and *C* is the arterial compliance. *Q*(s) was then scaled into its one-dimensional equivalent (rate of shortening) and used to control the rate of length change of the muscle. The ejection phase continued until the arterial pressure exceeded the ventricular pressure, whereupon the trabecula relaxed isometrically. As the rate of stress change approached zero, the trabecula was restretched back to its initial length. The restretch rate was user defined by a constant volumetric refill rate and scaled into the equivalent muscle length.

The FPGA calculated these parameter values at a rate of 20 kHz and applied the predicted rate of shortening to the trabecula in real time. The afterload generated during work-loops was adjusted by modifying *R_p_* within the range from 5 kPa·s/mL to 1,000 kPa·s/mL_,_ while *C* and *Z_c_* were held constant at 0.032 mL/kPa and 5 kPa·s/mL, respectively. These values were adapted from a previous study (22). The volumetric flow rate was set to 0.6 mL/s as this was sufficient for the restretch phase to complete within the diastolic period of each stress twitch at a stimulus frequency of 2 Hz and a temperature of 32°C.

### Preparing Trabeculae

To obtain trabeculae, 8- to 10-wk-old male Wistar rats were anesthetized with gaseous isoflurane (5% in O_2_) before being injected with heparin (1,000 IU/kg). The rats were killed via cervical dislocation and their hearts removed within 30 s. Each excised heart was immediately submerged in ice-cold 2,3-butanedione monoxime (BDM)-Tyrode’s solution consisting of (in mM): 130 NaCl, 6 KCl, 1.5 MgCl_2_, 0.5 NaH_2_PO_4_, 0.3 CaCl_2_, 10 glucose, and 20 2,3-butanedione monoxime (BDM). The heart was then transferred to a dissection bath, and Langendorff perfused with the BDM-Tyrode’s solution at room temperature with the pH adjusted to 7.4. The atria were removed and the ventricles opened by cutting along the septum. Trabeculae used within this study were excised from the right ventricle.

Each dissected trabecula was mounted between two platinum hooks in the cardiomyometer (20). Voice-coil motors mechanically coupled to each hook set the slack length of the trabecula. Platinum electrodes upstream and downstream of the measurement chamber were used to stimulate the trabecula electrically. The resultant stress produced by the trabecula was inferred from the deflection of a steel cantilever, with a known stiffness, attached to the downstream hook. When mounted in the cardiomyometer, the trabecula was superfused with Krebs-Henseleit (K-H) solution (in mM): 118 NaCl, 4.75 KCl, 1.18 MgSO_4_, 1.18 KH_2_PO_4_, 24.8 NaHCO_3_, 1.5 CaCl_2_, and 10 glucose. The superfusate was bubbled with carbogen (95% O_2_, 5% CO_2_) to maintain the pH at 7.4 and supply the muscle with ample oxygen throughout the protocol. The stimulus frequency was set to 1 Hz throughout an hour-long equilibration period for the acclimatization of the trabecula.

### Determining *L_100_*

After the stress generated by the trabecula had stabilized, when the diastolic and peak stress production had remained constant over a 10-min window, the muscle was considered to have acclimatized. It was then stretched to optimal length (*L_100_*). A central window was imaged with a ×20 objective lens, and the average sarcomere length was calculated using a two-dimensional (2-D)-fast Fourier transform algorithm (24). Trabeculae were discarded if it was not possible to image sarcomeres in a central region.

For each experiment, *L_100_* was defined as the muscle length corresponding to an average measured sarcomere length of ∼2.3 μm in the central region of each trabecula. *L_95_* was defined as 95% of the *L_100_* muscle length. Muscle length was maintained throughout each contraction using proportional-integral-derivative-controlled voice-coil motors informed by continuous measurement via laser interferometry.

### Ca^2+^ Transient Measurements

Fura-2 was used to measure intracellular Ca^2+^ dynamics in the trabeculae. Briefly, the broadband light produced by a 300 W xenon arc lamp was cyclically switched at 600 Hz between three different central wavelengths of 340 nm, 365 nm, and 380 nm, each with a bandwidth of 10 nm. The filtered light was projected onto a central portion of the trabecula via the bright-field microscope objective. The resultant fluorescent emission passed through a 510-nm bandpass filter before the intensity was measured using a photomultiplier tube. Before loading the trabecula with Fura-2, the background fluorescence of the trabecula was recorded. These autofluorescence measurements were subtracted from the fluorescence signals before calculating the ratio of the emission associated with the 340-nm and 380-nm excitation light (*F_340_/F_380_*). During loading, the stimulation frequency was set to 0.2 Hz, and the muscle superfused with a loading solution comprised K-H solution with 10 μM of cell-permeable Fura-2/AM and 1 mM probenecid. Loading was considered complete when *F_365_* had reached 10 times the background fluorescence measurement or the loading period had reached 2 h. Following loading, the superfusate was switched back to the standard K-H solution with 1 mM probenecid, and the stimulus frequency increased to 2 Hz. Intracellular Ca^2+^ dynamics were taken as *F_340_/F_380_*.

### Experimental Protocol

Following the achievement of a steady state of both stress production and the fluorescence ratio (unchanged diastolic and peak measurements over a 10-min period), the bath temperature was increased to 32°C using a thermoelectric heat pump in combination with a temperature controller. The experiment was performed at 32°C as a compromise between physiological temperature (37^o^C) and the Fura-2 leak rate. At 37°C, the leak rate of Fura-2 is substantial even in the presence of probenecid (25), an inhibitor of the anion transporters that drive the leakage. Lowering the experimental temperature to 32°C provided confidence that the entire preload-afterload protocol could be completed before extensive Fura-2 leakage occurred. This choice of temperature meant that the stimulation frequency had to be reduced to 2 Hz to provide an adequate diastolic period within which restretch could be completed before the start of the subsequent contraction.

After the temperature stabilized, the experiment commenced by setting the muscle to one of two preloads, achieved by setting the muscle length to either *L_95_* or *L_100_*. The selection of the first preload used was randomized for each trabecula. The trabecula was then stimulated to contract isometrically while the deflection of the force transducer was compensated for using the upstream motor, thereby maintaining muscle length. Once the isometric stress transient reached a beat-to-beat steady state, the muscle was subjected to FPGA-driven three-element Windkessel work-loops until stable loops were achieved. This process was repeated for six *R_p_* values ranging from 5 kPa·s/mL to 1,000 kPa·s/mL to characterize the afterload-dependent behavior between close to minimal (isotonic) and maximal (isometric) stress. The order in which *R_p_* values were used was randomized to mitigate the introduction of fatigue basis into the measurements. The muscle length was changed to *L_100_* or *L_95_* depending on the starting length, and the process was repeated until data for all afterloads had been collected. Force and Ca^2+^ data were collected for the duration of this protocol.

### Trabecula Dimension

At the end of the experiment, muscle geometry was measured at *L_100_* with the trabecula at quiescence. In total, seven trabeculae were studied. Their cross-sectional area was measured using spectral-domain optical coherence tomography as described previously (26). Briefly, a broadband (100 nm) low-coherence superluminescent diode with a central wavelength of 840 nm was split into a measurement and reference path. The reference path contained only a mirror, and the measurement path had a 2-D galvanometer that directed and focused the light onto the trabecula. The superposition of the back reflections from each optical path was passed through a spectrometer. The frequency domain encodes depth information in spectral-domain optical coherence tomography (27). After subtracting the background image, the interference pattern was inverse fast Fourier transformed. Steering the measurement arm along a single axis enabled the imaging of muscle cross section, and using a second axis, enabled the imaging of muscle volume. The resultant images were segmented, and the cross-sectional area was calculated by scaling the pixel area by the lateral and depth resolutions of the optical coherence tomography (OCT) (20). The seven trabeculae had an average cross-sectional area of 0.06 ± 0.04 mm^2^, an average length at *L_100_* of 2.2 ± 0.5 mm, and a major:minor width ratio of 1.2 ± 0.3 (values expressed as means ± SD). From the OCT images, we approximated the major and minor diameters of the trabeculae that represented the way an experimentalist may have been able to measure using a microscope and an angled mirror. These major and minor diameters averaged 300 ± 100 µm and 260 ± 40 µm, respectively.

### Possible Influence of Motion

It is important to consider the possibility that any changes to the Ca^2+^ transient may be a consequence of motion artifacts, as has been considered previously (7). Here, motion artifacts were investigated in a separate experiment using a gated imaging protocol (20). The imaging systems of the cardiomyometer are fixed at the center of the measurement chamber and have limited fields of view. Hence, to capture imaging data for the entire trabecula, it must be moved relative to the imaging systems. The upstream and downstream hooks can move in synchrony to achieve such a movement. By moving the trabecula to five different positions within the measurement chamber each 50 μm apart, we were able to perform gated imaging on an actively contracting trabecula (20). At each position, the trabecula performed work-loop contractions with the same loading conditions (i.e., preload and afterload) while bright-field images (100 fps) and stress and fluorescence data (20 kHz) were captured.

Following the experiment, the bright-field and fluorescence images were analyzed using a weighted averaging technique. First, the bright-field images from each window were stitched together and a central point in the most upstream imaging window was tracked using a subpixel registration algorithm (28). A measured Gaussian function of the regional fluorescence signal intensity was aligned with each bright-field imaging window. The tracking information was used to determine the relative signal intensity of the tracked point for each window at each time point. These signal intensities were normalized to the total sum to generate weighting factors and applied to the fluorescence signal from each window. The closer the tracked point was to the center of an imaging window, the greater the weighting factor for the fluorescence signal of that window. There was less than a 0.5% difference in the Ca^2+^ transient amplitude between the motion-compensated and uncompensated signals. Hence, the Ca^2+^ fluorescence signals reported here were not compensated for motion.

### Data Analysis

**Figure 1.**
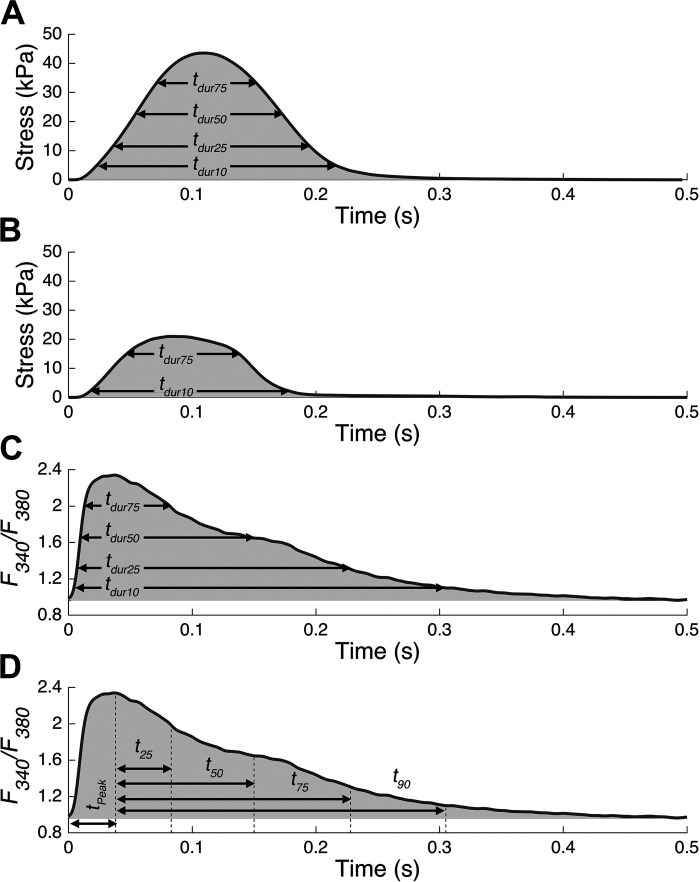
Twitch stress and Ca^2+^ transient parameters. Each panel shows a transient computed by averaging 10 steady-state stress twitches or Ca^2+^ transients. The passive component of stress twitches has been accounted for and only the active stress is shown. *A*: twitch duration measurements for an averaged isometric stress profile. *t_durx_* refers to the time that stress remained greater than *X* % of the peak active stress and was calculated for 10% (*t_dur10_*), 25% (*t_dur25_*), 50% (*t_dur50_*), and 75% (*t_dur75_*) of the stress amplitude. The shaded area represents the stress-time integral (STI). *B*: twitch duration measurements for an averaged work-loop contraction. For this work-loop twitch, the afterload was 0.31 (decimal fraction of the peak isometric stress) as achieved by setting *R_p_* to 50 kPa·s/mL. Four measures of twitch duration (as in *A*) were assessed, but only *t_dur10_* and *t_dur75_* have been included on the panel to mitigate visual clutter. The shaded area represents the integral of the twitch. *C*: twitch duration measurements for an averaged Ca^2+^ transient. *t_durx_* here refers to the time that the fluorescence signal remained greater than *X* % of the peak signal and was calculated for 10% (*t_dur10_*), 25% (*t_dur25_*), 50% (*t_dur50_*), and 75% (*t_dur75_*) of the Ca^2+^ transient amplitude. The *F_340_/F_380_*-time integral (CTI) is represented by the shaded area. *D*: decay time measurements for an averaged Ca^2+^ transient. *t_X_* equates to the time to *X* % decay from the peak fluorescence and was calculated for 25% (*t_25_*), 50% (*t_50_*), 75% (*t_75_*), and 90% decay (*t_90_*). The maximum rate of rise for stress and Ca^2+^ transients was also computed but have not been included on any panels.

Data were acquired using custom-written LabVIEW (National Instruments) software and analyzed offline using a series of custom-written MATLAB (MathWorks) programs. Muscle-specific averages of 10 steady-state force twitches and Ca^2+^ transients were computed for each preload and afterload, and various parameters describing active stress and Ca^2+^ dynamics were extracted (Fig. 1). Data would have been discarded if a trabecula experienced stress rundown of more than 20% for a single preload (29). However, no trabecula that underwent the entire protocol exhibited rundown to this extent. Active stress rundown per preload was 6.9% ± 5.3% (mean ± SD).

Muscle force was converted to stress using the measured cross-sectional area. Active twitch stress under the isometric contraction was revealed by subtracting the constant passive force from the total twitch stress (Fig. 1*A*). For work-loop contractions, the dynamic nature of the passive component of total stress during work-loop contractions meant that it was necessary to account for this dynamic effect when extracting parameters pertaining to active stress dynamics. This was achieved by first fitting a third-order polynomial to the passive stress-length trajectory of the lowest afterloaded work-loop. In combination with the fit results, the stored length information for a work-loop twitch was then used to estimate the dynamic passive force. Subtracting the resultant passive estimate from the total force isolated the active component for analysis (Fig. 1*B*).

The dynamics of stress were analyzed using the following parameters: stress-time integral (STI), maximum rate of stress development (*dS/dt*), and various twitch durations (*t_dur75_*, *t_dur50_*, *t_dur25_*, and *t_dur10_*). The twitch durations represent the time that stress remained greater than *X* % of active stress, where *X* is the subscript number (e.g., 25 in *t_dur25_*). Equivalent parameters were extracted to analyze the Ca^2+^ dynamics in the form of *F_340_/F_380_* time integral (CTI), maximum rate of *F_340_/F_380_* rise (*dF_340_/F_380_/dt*), and transient durations (*t_dur75_*, *t_dur50_*, *t_dur25_*, and *t_dur10_*). In addition, various decay timings (*t_25_*, *t_50_*, *t_75_*, and *t_90_*), time to peak (*t_peak_*), diastolic, peak, and *F_340_/F_380_* amplitude were calculated. The decay timings represent the time the Ca^2+^ signal takes to decay by *X* % from the peak (e.g., *t_25_* represents the time for the Ca^2+^ transient to decay by 25% from the peak *F_340_/F_380_* signal). With the exception of the rate of rise of stress and *F_340_*/*F_380_*, all parameters are illustrated in Fig. 1.

These parameters were plotted against relative end-systolic stress (ESS). ESS was defined as the stress at the time point where muscle length stopped reducing at the end of the shortening phase and represents the afterload experienced by the muscle. This was expressed relative to the isometric stress of the associated initial length. Hence, relative ESS was equal to 1 for isometric contractions.

### Statistical Analysis

An *F* test was performed for each parameter to determine whether a first- or second-order polynomial was sufficient to describe the relationship. The relationship included the parameters pertaining to twitch stress, and those pertaining to the Ca^2+^ transient, as functions of relative ESS. Statistical regression between the independent and dependent variables was performed using SAS software (Copyright 2021 SAS Institute Inc.). Linear mixed-effect models in SAS were used with muscle number treated as a random effect, and the initial muscle length (preload) and relative ESS (afterload) treated as fixed effects. A compound symmetry covariance structure was used in the model statement as this corresponded with the model with the smallest Akaike information criterion value. Afterload dependence was considered significant for each preload if the *P* value associated with either the linear term or, when applicable, the quadratic term, in the fixed effect between independent and dependent variables was <0.05. The effect of preload was considered significant if the *P* value associated with the “Contrast” statement in SAS output between the two regression lines for *L_100_* and *L_95_* was <0.05.

## RESULTS

Data were obtained to elucidate the effects of preload and afterload on the Ca^2+^ handling of cardiac muscle during isometric (Fig. 1*A*) and work-loop (Fig. 1*B*) contractions. These data consist of stress (Fig. 1, *A* and *B*) and Ca^2+^ (Fig. 1, *C* and *D*) information collected from seven right-ventricular trabeculae, each exposed to a series of work-loops at six different afterloads and two different initial muscle lengths of *L_100_* and *L_95_*. Several parameters were quantified from these average twitches and Ca^2+^ transients, including the timings annotated in Fig. 1. The average minimum relative ESS across the seven muscles determined the plotting range used for the linear regression for each preload.

**Figure 2.**
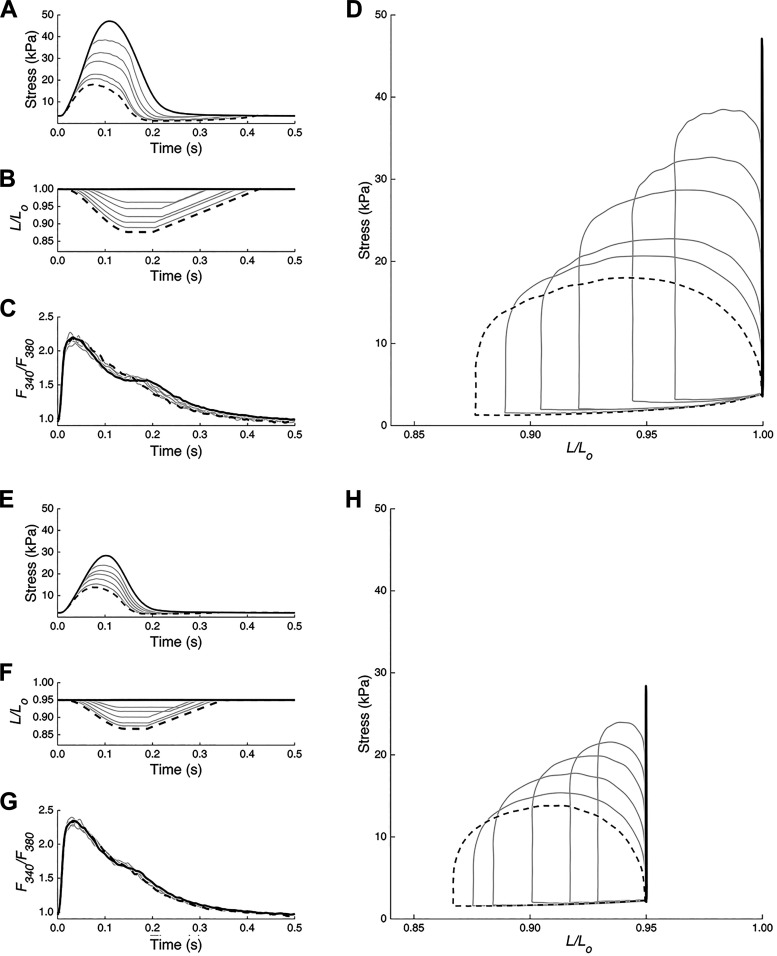
Stress-length work loops and Ca^2+^ transients of a representative trabecula at two different preloads. Steady-state twitch stress (*A* and *E*), muscle length (*B* and *F*), and Ca^2+^ transient (*C* and *G*) for a set of work loops over a range of afterloads at fixed preloads set by holding the muscle at *L_100_* (*A–D*) and *L_95_* (*E–H*). In all panels, twitches are overlaid. The solid dark line represents the isometric twitch. The dashed line represents the work-loop twitch at the lowest afterload. *D* and *H*: steady-state stress-length work loops are revealed when stress and length traces are plotted parametrically for each preload.

The stress, length, and *F_340_/F_380_* profiles from an exemplar trabecula at each preload are superimposed in Fig. 2, with the stress-length work-loops presented in Fig. 2, *D* and *H*. Increasing *R_p_* increased afterload and thereby increased ESS. Afterload did not appear to affect the amplitude of the Ca^2+^ transient but had effects on the duration of the Ca^2+^ transient (Fig. 2, *C* and *G*). The afterload-induced effects on the duration of Ca^2+^ transient appeared to be less pronounced at the reduced preload (Fig. 2*G* vs. Fig. 2*C*). A secondary component of the Ca^2+^ transient relaxation phase, or “bump,” appeared to be more pronounced during isometric contractions (Fig. 2, *C* and *G*) and at the greater preload (Fig. 2*C* vs. Fig. 2*G*). The isometric active stress at *L_100_* was 37 ± 17 kPa and the resting tension was 7 ± 4 kPa, which were significantly greater (*P* < 0.01) than those at *L_95_* (the isometric active stress was 23 ± 10 kPa and the resting tension was 3 ± 1 kPa; each presented as means ± SD).

**Figure 3.**
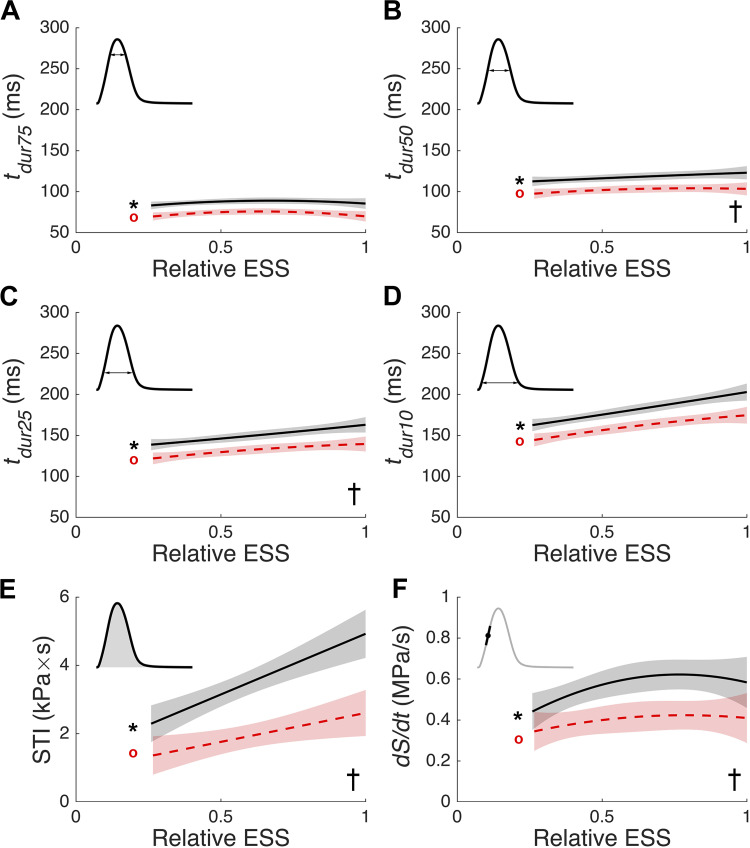
Preload and afterload dependence of the steady-state stress production. Data from 14 different experimental conditions on *n* = 7 muscle samples were analyzed to extract the following parameters: twitch durations (*A–D*), stress-time integral (STI; *E*), and the maximum rate of rise of stress development (*dS/dt*; *F*). The fixed-effect results of the linear mixed-effects model fitting are indicated for each of the two muscle lengths: *L_100_* is indicated with a solid black line, and *L_95_* is indicated with a dashed red line. The pale shading surrounding each fitted line indicates the 95% confidence band, calculated from the covariance matrix [as described previously (30)]. STI against relative ESS was fitted with a first-order polynomial; all other stress parameters were fitted with a second-order polynomial. These data are plotted against afterload, expressed as “relative ESS.” Each panel contains an inset that indicates how the respective metric was calculated. Data were analyzed using linear mixed-effect models implemented in SAS with muscle number treated as a random effect, and the initial muscle length (preload) and relative ESS (afterload) treated as fixed effects. *Significant afterload dependence at *L_100_*; red ^o^significant afterload dependence at *L_95_*; †significant preload dependence. ESS, end-systolic stress.

Figure 3 shows the effects of preload and afterload on stress production. At both preloads, all twitch duration metrics (*t_dur75,_ t_dur50,_ t_dur25_*, and *t_dur10_*) increased with increasing afterload (Fig. 3, *A*–*D*). The regression line between duration and relative ESS at *L_100_* was significantly different from that at *L_95_*, only for *t_dur50_* (Fig. 3*B*) and *t_dur25_* (Fig. 3*C*). Both the stress-time integral (STI) and maximum rate of rise of stress were afterload and preload dependent (Fig. 3, *E* and *F*). The afterload dependence of the maximum rate of rise of stress (*dS*/*dt*) reached a plateau at higher afterloads (Fig. 3*F*). Similarly, the time that peak *dS*/*dt* occurred followed a comparable trajectory to the afterload-dependence of maximal *dS*/*dt* (not shown). It is worth noting that the time at which *dS*/*dt* occurred always preceded the commencement of shortening in work-loop contractions.

**Figure 4.**
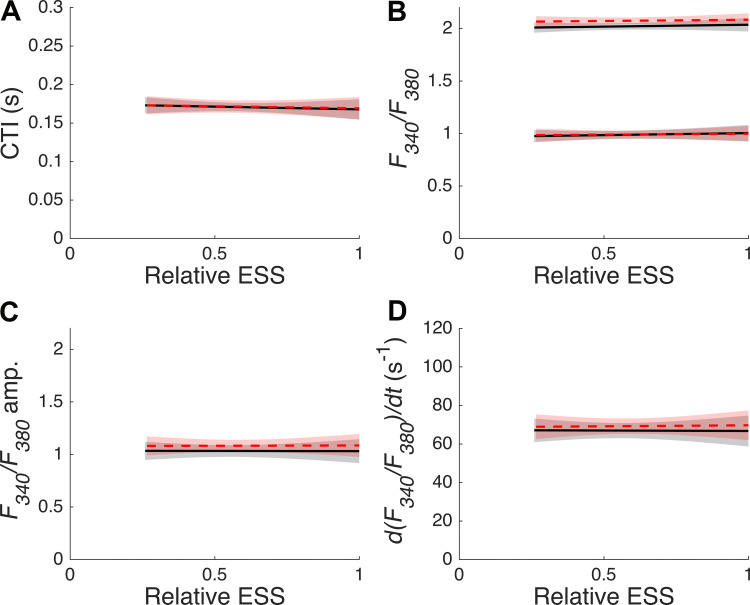
Preload and afterload dependence of Ca^2+^ transient. Data from 14 different experimental conditions on *n* = 7 muscle samples were analyzed to extract the following parameters: *F_340_/F_380_* time integral (CTI; *A*), peak (upper lines) and diastolic (lower lines) *F_340_/F_380_* signal (*B*), the *F_340_/F_380_* signal amplitude (*C*), and the maximum rate of rise of the *F_340_/F_380_* signal (*D*). The fixed-effect results of the linear mixed-effects model fitting for each of the two muscle lengths, *L_100_* is indicated with a solid black line and *L_95_* is indicated with a dashed red line. The pale shading surrounding each fit indicates the 95% confidence band, calculated from the covariance matrix [as described previously (30)]. These data are plotted against afterload, expressed as relative ESS. ESS, end-systolic stress. Data were analyzed using linear mixed-effect models implemented in SAS with muscle number treated as a random effect, and the initial muscle length (preload) and relative ESS (afterload) treated as fixed effects.

No statistically significant afterload or preload dependence was observed in the *F_340_/F_380_*-time integral (CTI), peak, diastolic, amplitude, or the maximum rate of rise of the *F_340_/F_380_* signal (Fig. 4, *A–D*, respectively).

**Figure 5.**
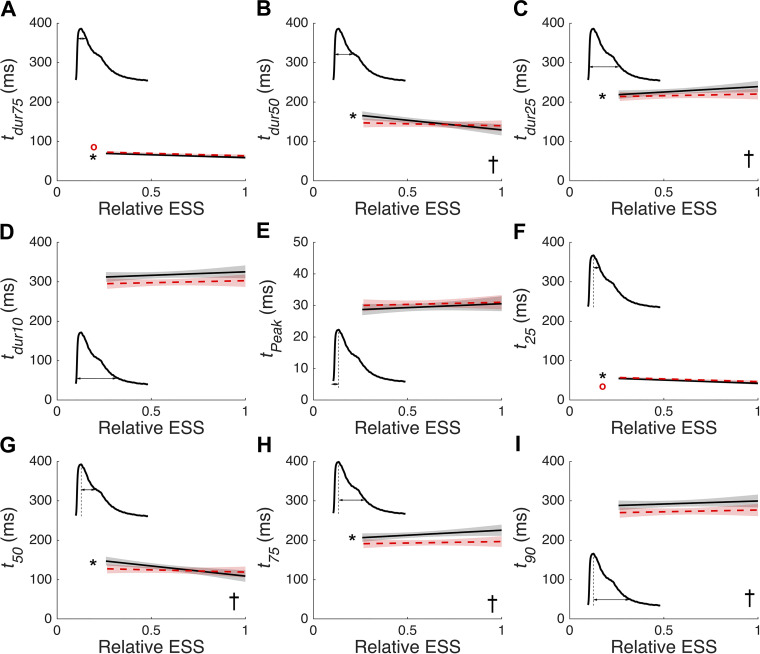
Preload and afterload dependence of the time course of Ca^2+^ transient. Data from 14 different experimental conditions on *n* = 7 muscle samples were analyzed to extract the following parameters: Ca^2+^ transient durations (*A–D*), time to *F_340_/F_380_* signal Ca^2+^ (*E*), and the time to *X* % decay from the peak *F_340_/F_380_* (*F–I*). The fixed-effect results of the linear mixed-effects model fitting for each of the two muscle lengths, *L_100_* is indicated with a solid black line and *L_95_* is indicated with a dashed red line. The pale shading surrounding each fit indicates the 95% confidence band, calculated from the covariance matrix [as described previously (30)]. These data are plotted against afterload (“relative ESS”). Each panel contains an inset that indicates how each metric was calculated. Data were analyzed using linear mixed-effect models implemented in SAS with muscle number treated as a random effect, and the initial muscle length (preload) and relative ESS (afterload) treated as fixed effects. *Significant afterload dependence at *L_100_*, red ^o^significant afterload dependence at *L_95_*, †significant preload dependence. ESS, end-systolic stress.

However, as suggested by the representative data in Fig. 2, *C* and *G*, some of the Ca^2+^ transient durations were afterload and preload dependent. Like the twitch duration metric, *t_dur25_* (Fig. 5*C*) increased with increasing afterload at *L_100_*. Yet, unlike in the stress case, both *t_dur75_* (Fig. 5*A*) and *t_dur50_* (Fig. 5*B*) correlated negatively with afterload, but *t_dur10_* was afterload independent (Fig. 5*D*). At *L_95_*, only *t_dur75_* was significantly afterload dependent. There was no significant preload dependence for *t_dur75_* or *t_dur10_*, but significant preload dependence for *t_dur50_* and *t_dur25_*. There was neither preload nor afterload dependence in the time to the peak of the Ca^2+^ transient (Fig. 5*E*). *t_dur50_* and *t_dur25_* and the equivalent decay parameters, *t_50_* and *t_25_*, aligned well with the start and end of the “bump” in the relaxation phase of the Ca^2+^ transient, respectively.

The timings associated with the decay of intracellular Ca^2+^ (*t_25_*, *t_50_*, *t_75_*, and *t_90_*) were plotted in Fig. 5, *F*–*I*. At *L_100_*, afterload dependence was obtained at the three earlier decay timings (Fig. 5, *F*–*H*) only. At *L_95_*, afterload dependence was obtained at *t_25_*, but not at *t_50_*, *t_75_*, or *t_90_*. Preload dependence was significant at all Ca^2+^ decay timings with the exception at *t_25_*. The initial negative relationship between Ca^2+^ decay timing and relative ESS became positive and eventually flat later within the decay phase.

## DISCUSSION

To our knowledge, this is the first study to have measured Ca^2+^ transients in isolated cardiac trabeculae under a work-loop contraction mode that mimics the contraction pattern of the whole heart. This represents the first novelty of this study, which has extended the contraction modes studied previously in understanding cardiac Ca^2+^ handling: afterloaded isotonic contraction (5), unloaded shortening (7), and sinusoidal length perturbation (31). The second novelty is the finding that the afterload dependence of the cardiac Ca^2+^ transient is preload dependent, which serves to reconcile conflicting afterload-dependent Ca^2+^ handling results in the literature. We evaluated the effects of preload and afterload on stress and Ca^2+^ dynamics by deriving various morphological and time course parameters (Fig. 1) as functions of relative ESS (Figs. 3, 4, and 5). Reducing preload from *L_100_* to *L_95_* diminished the afterload dependence of the Ca^2+^ transient timing, particularly during the decay phase.

### Experimental Design Considerations

In these experiments, the work-loop contraction mode was designed to simulate the pressure-volume loop typically observed in vivo (22), with the ability to vary the hemodynamic parameters governing the arterial impedance, the aortic compliance, and peripheral resistance. Isolated right-ventricular trabeculae were made to perform isometric and work-loop contractions over a range of afterloads at two preloads (Fig. 2) to assess their effects on the twitch stress and Ca^2+^ independently and simultaneously. The model parameters for the Windkessel work-loop contractions (*R_p_*, *Z_c_*, and *C*) were adapted from a previous study (22), in line with the in vivo measurements on the rat (32). Only *R_p_*, which represents the peripheral resistance, was changed in this study as previous work showed its dominant effect on afterload (22). We selected the range of *R_p_* values to encapsulate much of the afterload range between unloaded isotonic and isometric contractions.

### Effect of Preload and Afterload on Stress Production

Under Windkessel work-loop loading conditions, the measured stress dynamics aligned well with the existing literature. Twitch stress duration (Fig. 3, *A*–*D*) increased with afterload (3, 33, 34). STI (Fig. 3*E*), an index of cardiac energy expenditure, was enhanced for twitches at greater preloads and afterloads (35, 36). The maximum rate of rise of the stress (*dS*/*dt*; Fig. 3*F*) was greater at longer muscle lengths and higher afterloads for both muscle lengths. As afterload increases, *dS*/*dt* reached a plateau at both preloads. This suggests that the limiting factor for *dS*/*dt* is the timing of the onset of shortening, which increases with afterload. These results are unsurprising as, for a given preload, the twitch profile will follow the same isometric upstroke until the stress produced is sufficient to overcome the external afterload and initiate muscle shortening. Existing studies unequivocally support this preload (37, 38) and afterload dependence (36, 37, 39). Therefore, using Windkessel work loops did not modify the expected preload- and afterload-dependent stress dynamics consistently observed in the literature.

### Effect of Preload and Afterload on Ca^2+^ Handling

Confirming the consistency between our data on the stress dynamics with the literature outlined above enables the consideration of the effects of preload and afterload on Ca^2+^ transients. With the use of an isometric contraction protocol, a length-independent Ca^2+^ amplitude reminiscent of our data (Fig. 4*C*) has been reported previously (40, 41). However, such a result is not ubiquitous (13, 42, 43). The measurement of length-dependent Ca^2+^ transients may be confounded by the slow-force response of cardiac muscle to sudden length changes, which can induce a transitory modification of the Ca^2+^ transient (44, 45).

The afterload dependence of the Ca^2+^ transient is contentious. Although numerous studies observed a null effect of afterload on the Ca^2+^ transient (6, 8, 31), two studies have reported a negative dependence of Ca^2+^ transient amplitude on afterload (5, 7). The substantial afterload-dependent augmentation of the Ca^2+^ transient seen in trabeculae from failing hearts was much smaller in healthy human trabeculae (5). In addition, Yasuda et al. (7), the other study that observed an acute modification phenomenon, performed a unique experimental protocol of switching between isotonic and isometric every few twitches (7). In our experiment, contraction mode was changed after the stress development and Ca^2+^ amplitude had reached a steady state.

Considering the afterload dependence of Ca^2+^ dynamics at *L_100_*, only the timing of the transients displayed significant dependence, particularly in the decay phase (Fig. 5). Afterload-dependent Ca^2+^ decay aligns well with both modeling (10–12) and experimental (5, 7, 40) work that studied afterload-dependent Ca^2+^ handling only.

The afterload-dependent behavior observed in the Ca^2+^ transient is well explained by force-modulated cooperativity of Ca^2+^-TnC binding (40, 46). *T_25_* represents the initial decay of the Ca^2+^ transient, and it increased as the afterload decreased (Fig. 5*F*). Initial Ca^2+^ decline occurs during the upstroke of the force twitch (Fig. 2), where free intracellular Ca^2+^ concentration decreases due to sequestering mechanisms and Ca^2+^ binding to TnC. If the phosphorylation state of the myofilaments is constant with afterload, the reduction of free intracellular Ca^2+^ associated with TnC binding would be proportional to force; the greater the force, the more Ca^2+^ bound to TnC, i.e., cooperativity. Hence, the slower initial decay of Ca^2+^ transients observed at lower afterloads was as expected, largely in line with the force-modulated cooperativity mechanism.

The plateau in the Ca^2+^ transient decay phase, or “bump,” is a morphological feature that has been observed in several animal studies (16, 46, 47) and predicted, to some extent, in silico (12). The bump is thought to be caused by Ca^2+^ dissociation from troponin-C (TnC) during relaxation at a faster rate than it can be sequestered into the sarcoplasmic reticulum (SR) (46). Jiang et al. (46) confirmed that the amplitude of the bump is correlated with the number of bound cross bridges and that the start time of the bump depends on the stress twitch duration. As the twitch duration increased, the bump occurred later in the Ca^2+^ transient. Our results at *L_100_* are consistent with the proposed mechanism. At higher afterloads, the overall duration (*t_dur10_*) of the twitch stress increased. As expected, *t_50_*, which aligns approximately with the bump onset, decreased with increasing afterload, reflecting the delayed bump onset. Further, *t_75_*, which aligns with the bump region, was longer with increasing afterload, reflecting the enhanced bump amplitude. The final metric of the Ca^2+^ decay, *t_90_*, is not afterload dependent, and thus it appears that the afterload-induced TnC binding affinity does not affect the overall decay phase of the Ca^2+^ transient.

This is the first study to show, experimentally, some preload-induced modification of Ca^2+^ transient afterload dependency. We found that the decay-related timing metrics *t_50_*, *t_75_*, and *t_90_* had preload-informed modulation of their afterload dependency. The underlying molecular basis for this warrants further study, but we speculate that it too can be explained by the force-dependent Ca^2+^-TnC cooperativity. This speculation stems from two of our findings. First, the preload was more influential on the overall duration of the Ca^2+^ transient than afterload, as *t_90_* and *t_dur10_* (Ca^2+^) were shorter at the lower preload while being afterload independent (Fig. 5*I*). On initial inspection, the preload dependency of the Ca^2+^ transient duration measured within this study directly contradicts preexisting observations that greater muscle lengths are associated with shorter Ca^2+^ transients (48, 49) and with those reporting no length dependence (13). However, the observed behavior seems to align with a force-based binding affinity of TnC (50, 51). At the greater preload, more cross bridges can form, increasing the binding affinity of TnC for Ca^2+^, slowing the eventual Ca^2+^ dissociation (52), thereby extending the duration of the Ca^2+^ transient. Second, greater muscle lengths are associated with an augmented bump area (47) as preload modulates the amount of Ca^2+^ that will bind to TnC due to a greater number of strong-binding, force-producing, cross bridges. At lower preloads, the maximum area of the bump would be reduced, and therefore the previously described afterload-dependent modification of the bump would be less detectable. Hence, we suggest that the preload- and afterload-dependent modifications to the Ca^2+^ transient can be explained by force-dependent TnC binding affinity for Ca^2+^.

Given that the proposed mechanism for contraction-mode-dependent behavior relies solely on a dynamic Ca^2+^-TnC binding affinity, it follows that metrics describing Ca^2+^ release would not demonstrate mode dependence. As expected, the maximum rate of rise of the Ca^2+^ transient was unaffected by preload and afterload (Fig. 4*D*), as observed previously (6, 33). The time to peak Ca^2+^ was also independent of preload and afterload (Fig. 5*E*). In the literature, the time to peak Ca^2+^ is length independent in healthy cardiac muscle (13, 47, 48), and the afterload independence is also consistent with data collected from small- to medium-sized mammalian heart tissues (3, 4, 8). We found that the integral of the Ca^2+^ transient (CTI), which provides an insight into the exposure of internal structures to Ca^2+^, was independent of afterload and preload (Fig. 4*A*). This finding aligns well with data collected under similar experimental conditions (6, 8, 31).

Our results reconcile literature findings wherein the cases that observed no afterload dependence used equivalent muscle lengths much shorter than *L_100_* (6, 8) or only exposed preparations to high afterloads where the difference would be difficult to detect (31).

### Ca^2+^-Stress Decoupling

Stress and Ca^2+^ dynamics had distinct dependences on preload and afterload (Fig. 3 vs. Fig. 5). Cardiac Ca^2+^-stress decoupling is well known, even in systems with sudden reductions of muscle length (40, 46). Mechanistically, this is unsurprising given that the intracellular Ca^2+^ and the myofilaments only reach dynamic equilibrium at the end of diastole (53). Stress production is enhanced at *L_100_* from *L_95_* due to myofilament length-dependent activation underlined by various interactions including, but not limited to, more optimal myofilament overlap (54), reduced interfilament lattice spacing (55, 56), and enhanced sensitivity of the myofilament to Ca^2+^ (57, 58) that is associated with reduced phosphorylation state of TnI (59). Ca^2+^ transients, on the other hand, are dictated by the release and reuptake rates of Ca^2+^, which are dependent on intracellular Ca^2+^ concentration (60). A limitation of fluorescent dyes is that they measure the free intracellular Ca^2+^ (i.e., not bound to TnC). When the muscle shortens during contractions, the affinity for Ca^2+^ decreases and causes some Ca^2+^ to unbind from TnC and “slow” the Ca^2+^ transient decay (61). The complex interplay between the two dynamic systems of the Ca^2+^ transient and the force-producing cross-bridge cycle means that only a minor deviation in the Ca^2+^ transient is necessary to result in the substantial preload- and afterload-dependent twitch stress durations observed.

### Ca^2+^ Indicator Considerations

To measure intracellular Ca^2+^ handling in this study, we used the ratiometric fluorescent dye Fura-2 (62). An improper fluorescence loading procedure can result in incomplete acetyl-methyl ester (AM) hydrolysis of Fura-2/AM (63, 64) and subcompartmental loading of the dye into organelles (65, 66). Each of these issues can lead to erroneous fluorescence signals that are not correlated with cytosolic Ca^2+^ concentration. In this study, loading was performed at room temperature and Pluronic F-127 was included in the loading solution to ameliorate these issues, the success of which has been shown previously (67).

It has been suggested that, even at low intracellular concentrations, the rate constants of Fura-2 (*k_on_* and *k_off_*) are too low for the binding reaction of Fura-2 and Ca^2+^ to be at equilibrium during a Ca^2+^ transient (68). Should this be the case, one would expect some distortion of the Ca^2+^ transient morphology, beyond the nonlinear scaling required to transform *F_340_*/*F_380_* to [Ca^2+^]*_i_* (62). As each muscle was exposed to every combination of loading (seven afterloads each at the two preloads) and the order was randomized, these distortions could not have introduced bias into the investigation of load dependence.

The development of novel thin-filament localized sensors has created a new pathway for studying intracellular mechanisms. Sparrow et al. (69) describe the development of a genetically encoded fluorescent Ca^2+^ sensor that is localized to the thin filament. Similar to the fluorescent dyes outlined above, this sensor introduces buffering effects, which may disrupt force production, but has the major advantage of specifically targeting the troponin complex. Should our hypothesis hold true, force-dependent binding rates of Ca^2+^ to Tn should be observed. In addition, Vetter et al. (70) describe a genetically encoded fluorescent sensor that detects, in real time, the distance between TnI and TnC, which can be used to infer when the thin filament is active. Aligned with our hypothesis, loading their samples changed the Tn complex activation twitch, particularly in the duration of the activated period. Their preliminary findings align well with our proposed mechanism and it would be interesting to test explicitly using these technologies.

### Contraction-Mode Dependence

The end-systolic stress-length relation under the work-loop contraction mode is lower than that obtained under the isometric contraction mode (1, 2). Whereas recent modeling work predicts some afterload-dependent modifications of the Ca^2+^ transient decay phase (10–12), Shimizu et al. (8) replicated the stress-length profile for isometric and work-loop contractions using a contraction-mode-independent Ca^2+^ transient without including length-dependent myofilament Ca^2+^ sensitivity. This study, therefore, provides the required experimental data for modeling work to reconcile the Ca^2+^ transient with the contraction-mode dependence observed in the stress-length domain.

### The Physiological Relevance of Isometric Ca^2+^

It is more common for cardiac physiologists to study Ca^2+^ handling under isometric contraction conditions at *L_100_* than contraction modes that allow muscle shortening. Our results comparing these contraction modes raise questions regarding the suitability of using Ca^2+^ measurements under isometric conditions to infer those underlying work-loop contractions. At *L_100_*, there were significant differences in the decay phase of the Ca^2+^ transients when comparing work-loop and isometric contractions (Fig. 5, *F*–*H*). Thus, the isometric and work-loop Ca^2+^ transients at *L_100_* are not equivalent. The afterload dependence of the Ca^2+^ decay is modulated by preload, and, at *L_95_*, only *t_25_* was afterload dependent. With the results presented in this study, it would seem reasonable to discourage the study of Ca^2+^ handling under isometric conditions for muscle lengths greater than *L_95_*.

### Limitations

The working range of sarcomeres in an ex vivo heart can range from 1.9 µm to 2.4 µm (71–73). Here, trabeculae were studied at and near the optimal muscle length (sarcomere length of 2.3 µm) to replicate conditions under which ex vivo tissue experiments are commonly performed. As the afterload dependence of Ca^2+^ transients was assessed at only two preloads at *L_100_* (sarcomere length of 2.3 µm) and *L_95_*, it is difficult to extrapolate the afterload dependence to even lower preloads. At muscle lengths shorter than *L_85_*, we have observed that work-loop contractions result in out-of-plane motion where the distance between the trabecula and the microscope objective varied. To avoid this out-of-plane motion confounding the fluorescence signals, we limited the shortest muscle length to *L_95_* as the muscle shortening was ∼10% of the initial muscle length at the lowest afterload (Fig. 2*D*). Previous mechanistic studies have shown a substantial change in the contraction-mode dependence of the stress-length relation between *L_100_* and *L_95_* (2). Hence, we were confident that, should the Ca^2+^ handling be preload dependent, any dependency would be detectable with the two selected preloads. Extrapolating the contraction-mode dependence of the stress-length relation, one could speculate that the afterload dependence of Ca^2+^ transients would continue to diminish as preload is reduced. Perhaps the afterload dependence would be eliminated at the muscle lengths corresponding to the functional sarcomere length in vivo of 2.1 μm (74) or 90% to 95% of the optimal length at physiological end-diastolic pressures (71). A future study may involve the investigation of muscle lengths greater than *L_100_* or under interventions that mimic conditions of physiological stress and exercise.

Isometric force control in this study was achieved by maintaining muscle length rather than sarcomere length. We did not measure internal shortening explicitly during this study, though a previous study indicates that sarcomere length may not be homogenous during fixed-end contraction at *L_100_* (28). To control the muscle length such that the sarcomere length in the central region is fixed would require stretching the sarcomeres at each end of the trabecula. Furthermore, Janssen and de Tombe (75) showed that uncontrolled sarcomere shortening during isometric contractions, as is the case in muscle length-based control, changes the amplitude of the Ca^2+^ transient but not the timing of Ca^2+^ transients. Thus, we believe that muscle-length control during the isometric phases of the implemented work-loop contraction protocol has negligible impacts on the parameters quantifying the time course of the Ca^2+^ transient, which we found were afterload and preload dependent.

### Conclusions

These experiments exploiting work-loop contractions demonstrate that the parameters associated with Ca^2+^ transients display a mixed dependence on afterload and preload. Whereas the amplitude, integral and rates are independent of afterload and preload, the decay-phase time courses are afterload dependent, an effect augmented at the greater preload. These results show a preload dependence to the afterload dependence of the cardiac Ca^2+^ transient and serve to reconcile conflicting literature conclusions regarding whether Ca^2+^ transient is afterload dependent. This study provides ample evidence to discourage the common use of Ca^2+^ transients under isometric conditions to infer those under work-loop contractions.

## GRANTS

This study was funded by Doctoral Scholarships from The University of Auckland (awarded to J.M.D. and A.S.G.), Sir Charles Hercus Health Research Fellowships (20/011 and 21/116) from the Health Research Council of New Zealand (awarded to J-C.H. and K.T., respectively), a Doctoral Scholarship awarded by the Heart Foundation of New Zealand (awarded to A.A.), Marsden Fast-Start grants (UOA1504 and UOA1703) from the Royal Society of New Zealand (awarded to J-C.H. and K.T., respectively), and a James Cook Research Fellowship from the Royal Society of New Zealand (awarded to A.J.T.). The original development of this instrument was funded by a Marsden grant (11-UOA-199) from the Royal Society of New Zealand (awarded to A.J.T. and P.M.F.N.).

## DISCLOSURES

No conflicts of interest, financial or otherwise, are declared by the authors.

## AUTHOR CONTRIBUTIONS

J.M.D., K.T., A.S.G., A.J.A., P.M.F.N., A.J.T., and J-C.H. conceived and designed research; J.M.D. performed experiments; J.M.D. and J-C.H. analyzed data; J.M.D. interpreted results of experiments; J.M.D. prepared figures; J.M.D. drafted manuscript; J.M.D., K.T., A.S.G., A.J.A., P.M.F.N., A.J.T., and J-C.H. edited and revised manuscript; J.M.D., K.T., A.S.G., A.J.A., P.M.F.N., A.J.T., and J-C.H. approved final version of manuscript.

## References

[1] Han J-C, Loiselle D, Taberner A, Tran K. Re-visiting the Frank-Starling nexus. Prog Biophys Mol Biol 159: 10–21, 2021. doi:10.1016/j.pbiomolbio.2020.04.003. 32407748

[2] Han J-C, Pham T, Taberner AJ, Loiselle DS, Tran K. Solving a century-old conundrum underlying cardiac force-length relations. Am J Physiol Heart Circ Physiol 316: H781–H793, 2019. doi:10.1152/ajpheart.00763.2018. 30707611

[3] Housmans PR, Lee NKM, Blinks JR. Active shortening retards the decline of the intracellular calcium transient in mammalian heart muscle. Science 221: 159–161, 1983. doi:10.1126/science.6857274. 6857274

[4] Lab MJ, Allen DG, Orchard CH. The effects of shortening on myoplasmic calcium concentration and on the action potential in mammalian ventricular muscle. Circ Res 55: 825–829, 1984. doi:10.1161/01.RES.55.6.825. 6499137

[5] Vahl CF, Bonz A, Timek T, Hagl S. Intracellular calcium transient of working human myocardium of seven patients transplanted for congestive heart failure. Circ Res 74: 952–958, 1994. doi:10.1161/01.RES.74.5.952. 8156642

[6] White E, Boyett MR, Orchard CH. The effects of mechanical loading and changes of length on single guinea‐pig ventricular myocytes. J Physiol 482: 93–107, 1995. doi:10.1113/jphysiol.1995.sp020502. 7730993 PMC1157756

[7] Yasuda S-i, Sugiura S, Yamashita H, Nishimura S, Saeki Y, Momomura S-i, Katoh K, Nagai R, Sugi H. Unloaded shortening increases peak of Ca^2+^ transients but accelerates their decay in rat single cardiac myocytes. Am J Physiol Heart Circ Physiol 285: H470–H475, 2003. doi:10.1152/ajpheart.00012.2003. 12714336

[8] Shimizu J, Todaka K, Burkhoff D. Load dependence of ventricular performance explained by model of calcium-myofilament interactions. Am J Physiol Heart Circ Physiol 282: H1081–H1091, 2002. doi:10.1152/ajpheart.00498.2001. 11834507

[9] Balakina-Vikulova NA, Panfilov A, Solovyova O, Katsnelson LB. Mechano-calcium and mechano-electric feedbacks in the human cardiomyocyte analyzed in a mathematical model. J Physiol Sci 70: 12, 2020. doi:10.1186/s12576-020-00741-6. 32070290 PMC7028825

[10] Iribe G, Kaneko T, Yamaguchi Y, Naruse K. Load dependency in force-length relations in isolated single cardiomyocytes. Prog Biophys Mol Biol 115: 103–114, 2014. doi:10.1016/j.pbiomolbio.2014.06.005. 24976617

[11] Balakina-Vikulova N, Katsnelson L. Work performance in failing myocardium assessed in a mathematical model of the human ventricular myocyte electromechanical coupling. 2021 IEEE Ural-Siberian Conference on Computational Technologies in Cognitive Science, Genomics and Biomedicine (CSGB). Novosibirsk-Yekaterinburg, Russia, 2021. doi:10.1109/CSGB53040.2021.9496049.

[12] Guidry ME, Nickerson DP, Crampin EJ, Nash MP, Loiselle DS, Tran K. Insights from computational modeling into the contribution of mechano-calcium feedback on the cardiac end-systolic force-length relationship. Front Physiol 11: 587, 2020. doi:10.3389/fphys.2020.00587. 32547426 PMC7273927

[13] Han J-C, Tran K, Taberner AJ, Nickerson DP, Kirton RS, Nielsen PMF, Ward M-L, Nash MP, Crampin EJ, Loiselle DS. Myocardial twitch duration and the dependence of oxygen consumption on pressure-volume area: experiments and modelling. J Physiol 590: 4603–4622, 2012. doi:10.1113/jphysiol.2012.228965. 22570375 PMC3477760

[14] Lookin O. The use of Ca^2+^-transient to evaluate Ca^2+^ utilization by myofilaments in living cardiac muscle. Clin Exp Pharmacol Physiol 47: 1824–1833, 2020. doi:10.1111/1440-1681.13376. 32654202

[15] Lookin ON, Protsenko YL. The kinetics of cytosolic calcium in the right ventricular myocardium of guinea pigs and rats. Biophysics (Oxf) 61: 119–132, 2016. doi:10.1134/S0006350916010140.

[16] Lookin O, Protsenko Y. Length-dependent activation of contractility and Ca^2+^-transient kinetics in auxotonically contracting isolated rat ventricular cardiomyocytes. Front Physiol 10: 1473, 2019. doi:10.3389/fphys.2019.01473. 31920687 PMC6917588

[17] Schick BM, Dlugas H, Czeiszperger TL, Matus AR, Bukowski MJ, Chung CS. Reduced preload increases mechanical control (strain-rate dependence) of relaxation by modifying myosin kinetics. Arch Biochem Biophys 707: 108909, 2021. doi:10.1016/j.abb.2021.108909. 34015323 PMC8635462

[18] Iribe G, Helmes M, Kohl P. Force-length relations in isolated intact cardiomyocytes subjected to dynamic changes in mechanical load. Am J Physiol Heart Circ Physiol 292: H1487–H1497, 2007. doi:10.1152/ajpheart.00909.2006. 17098830

[19] Khokhlova A, Konovalov P, Iribe G, Solovyova O, Katsnelson L. The effects of mechanical preload on transmural differences in mechano-calcium-electric feedback in single cardiomyocytes: experiments and mathematical models. Front Physiol 11: 171, 2020. doi:10.3389/fphys.2020.00171. 32256377 PMC7091561

[20] Dowrick JM, Anderson AJ, Cheuk ML, Tran K, Nielsen PMF, Han J-C, Taberner AJ. Simultaneous brightfield, fluorescence, and optical coherence tomographic imaging of contracting cardiac trabeculae ex vivo. J Vis Exp 176: e62799, 2021. doi:10.3791/62799. 34661582

[21] Taberner A, Nielsen P, Johnston C, Anderson A, Cheuk ML, Garrett A, Dowrick JM, Tang ELP, Hajirassouliha A, Ruddy B, Pham T, Tran K, Han J-C, Loiselle D. A dynamometer for nature’s engines. IEEE Instrum Meas Mag 22: 10–16, 2019. doi:10.1109/MIM.2019.8674628.

[22] Garrett AS, Pham T, Loiselle D, Han J-C, Taberner A. Mechanical loading of isolated cardiac muscle with a real-time computed Windkessel model of the vasculature impedance. Physiol Rep 7: e14184, 2019. doi:10.14814/phy2.14184. 31512409 PMC6739510

[23] Seymour RS, Blaylock AJ. The principle of laplace and scaling of ventricular wall stress and blood pressure in mammals and birds. Physiol Biochem Zool 73: 389–405, 2000. doi:10.1086/317741. 11009393

[24] Lam Po Tang E, Laven RC, Hajirassouliha A, Nielsen PMF, Taberner AJ. Measurement of displacement in isolated heart muscle cells using markerless subpixel image registration. 2019 IEEE International Instrumentation and Measurement Technology Conference. Auckland, New Zealand, 2019. doi:10.1109/I2MTC.2019.8826940.

[25] Roe MW, Lemasters JJ, Herman B. Assessment of Fura-2 for measurements of cytosolic free calcium. Cell Calcium 11: 63–73, 1990. doi:10.1016/0143-4160(90)90060-8. 2191782

[26] Cheuk ML, Anderson AJ, Han J-C, Lippok N, Vanholsbeeck F, Ruddy BP, Loiselle DS, Nielsen PMF, Taberner AJ. Four-dimensional imaging of cardiac trabeculae contracting in vitro using gated OCT. IEEE Trans Biomed Eng 64: 218–224, 2017. doi:10.1109/TBME.2016.2553154. 27093310

[27] Lippok N, Coen S, Nielsen P, Vanholsbeeck F. Dispersion compensation in Fourier domain optical coherence tomography using the fractional Fourier transform. Opt Express 20: 23398–23413, 2012. doi:10.1364/oe.20.023398. 23188304

[28] Cheuk ML, Lam Po Tang E, HajiRassouliha A, Han J-C, Nielsen PMF, Taberner AJ. A method for markerless tracking of the strain distribution of actively contracting cardiac muscle preparations. Exp Mech 61: 95–106, 2021. doi:10.1007/s11340-020-00646-w.

[29] Kumar M, Govindan S, Zhang M, Khairallah RJ, Martin JL, Sadayappan S, De Tombe PP. Cardiac myosin-binding protein C and troponin-I phosphorylation independently modulate myofilament length-dependent activation. J Biol Chem 290: 29241–29249, 2015. doi:10.1074/jbc.M115.686790. 26453301 PMC4705930

[30] Feldman HA. Families of lines: random effects in linear regression analysis. J Appl Physiol (1985) 64: 1721–1732, 1988. doi:10.1152/jappl.1988.64.4.1721. 3379003

[31] Layland J, Kentish JC. Effects of α1‐or β‐adrenoceptor stimulation on work‐loop and isometric contractions of isolated rat cardiac trabeculae. J Physiol 524: 205–219, 2000. doi:10.1111/j.1469-7793.2000.t01-1-00205.x. 10747193 PMC2269858

[32] Molino P, Cerutti C, Julien C, Cuisinaud G, Gustin MP, Paultre C. Beat-to-beat estimation of windkessel model parameters in conscious rats. Am J Physiol Heart Circ Physiol 274: H171–H177, 1998. doi:10.1152/ajpheart.1998.274.1.H171. 9458865

[33] Han J-C, Taberner AJ, Nielsen PMF, Loiselle DS. Interventricular comparison of the energetics of contraction of trabeculae carneae isolated from the rat heart. J Physiol 591: 701–717, 2013. doi:10.1113/jphysiol.2012.242719. 23184511 PMC3577548

[34] Han J-C, Tran K, Nielsen PMF, Taberner AJ, Loiselle DS. Streptozotocin-induced diabetes prolongs twitch duration without affecting the energetics of isolated ventricular trabeculae. Cardiovasc Diabetol 13: 79, 2014. doi:10.1186/1475-2840-13-79. 24731754 PMC4005834

[35] Han J-C, Taberner AJ, Tran K, Nickerson DP, Nash MP, Nielsen PMF, Crampin EJ, Loiselle DS. Relating components of pressure-volume area in Suga’s formulation of cardiac energetics to components of the stress-time integral. J Appl Physiol (1985) 113: 988–995, 2012. doi:10.1152/japplphysiol.00438.2012. 22837173 PMC3487493

[36] Pham T, Nisbet L, Taberner A, Loiselle D, Han J-C. Pulmonary arterial hypertension reduces energy efficiency of right, but not left, rat ventricular trabeculae. J Physiol 596: 1153–1166, 2018. doi:10.1113/JP275578. 29363144 PMC5878219

[37] Konishi T, Nakamura Y, Kato I, Kawai C. Dependence of peak dP/dt and mean ejection rate on load and effect of inotropic agents on the relationship between peak dP/dt and left ventricular developed pressure—assessed in the isolated working rat heart and cardiac muscles. Int J Cardiol 35: 333–341, 1992. doi:10.1016/0167-5273(92)90231-Q. 1612796

[38] Sys SU, Brutsaert DL. Determinants of force decline during relaxation in isolated cardiac muscle. Am J Physiol Heart Circ Physiol 257: H1490–H1497, 1989. doi:10.1152/ajpheart.1989.257.5.h1490. 2589505

[39] Han J-C, Guild S-J, Pham T, Nisbet L, Tran K, Taberner AJ, Loiselle DS. Left-ventricular energetics in pulmonary arterial hypertension-induced right-ventricular hypertrophic failure. Front Physiol 8: 1115, 2017. doi:10.3389/fphys.2017.01115. 29375394 PMC5767264

[40] Backx PH, Ter Keurs HE. Fluorescent properties of rat cardiac trabeculae microinjected with fura-2 salt. Am J Physiol Heart Circ Physiol 264: H1098–H1110, 1993. doi:10.1152/ajpheart.1993.264.4.h1098. 8476086

[41] Lookin O, Balakin A, Kuznetsov D, Protsenko Y. The length-dependent activation of contraction is equally impaired in impuberal male and female rats in monocrotaline-induced right ventricular failure. Clin Exp Pharmacol Physiol 42: 1198–1206, 2015. doi:10.1111/1440-1681.12471. 26234534

[42] Kentish JC, Wrzosek A. Changes in force and cytosolic Ca^2+^ concentration after length changes in isolated rat. J Physiol 506: 431–444, 1998. doi:10.1111/j.1469-7793.1998.431bw.x. 9490870 PMC2230716

[43] Vahl CF, Timek T, Bonz A, Fuchs H, Dillman R, Hagl S. Length dependence of calcium- and force-transients in normal and failing human myocardium. J Mol Cell Cardiol 30: 957–966, 1998. doi:10.1006/jmcc.1998.0670. 9618236

[44] Dowrick JM, Tran K, Loiselle DS, Nielsen PMF, Taberner AJ, Han J-C, Ward M-L. The slow force response to stretch: controversy and contradictions. Acta Physiol (Oxf) 226: e13250, 2019. doi:10.1111/apha.13250. 30614655

[45] Schotola H, Sossalla ST, Renner A, Gummert J, Danner BC, Schott P, Toischer K. The contractile adaption to preload depends on the amount of afterload. ESC Heart Fail 4: 468–478, 2017. doi:10.1002/ehf2.12164. 29154423 PMC5695189

[46] Jiang Y, Patterson MF, Morgan DL, Julian FJ. Basis for late rise in fura 2 R signal reporting [Ca^2+^]_i_ during relaxation in intact rat ventricular trabeculae. Am J Physiol Cell Physiol 274: C1273–C1282, 1998. doi:10.1152/ajpcell.1998.274.5.c1273. 9612214

[47] Lookin O, Kuznetsov D, Protsenko Y. Sex differences in stretch-dependent effects on tension and Ca^2+^ transient of rat trabeculae in monocrotaline pulmonary hypertension. J Physiol Sci 65: 89–98, 2015. doi:10.1007/s12576-014-0341-8. 25359385 PMC10718032

[48] Allen DG, Kurihara S. The effects of muscle length on intracellular calcium transients in mammalian cardiac muscle. J Physiol 327: 79–94, 1982. doi:10.1113/jphysiol.1982.sp014221. 7120151 PMC1225098

[49] Komukai K, Kurihara S. Effect of developed tension on the time courses of Ca^2+^ transients and tension in twitch contraction in ferret myocardium. Cardiovasc Res 32: 384–390, 1996. doi:10.1016/0008-6363(96)00084-3. 8796126

[50] Hibberd MG, Jewell BR. Calcium- and length-dependent force production in rat ventricular muscle. J Physiol 329: 527–540, 1982. doi:10.1113/jphysiol.1982.sp014317. 7143258 PMC1224794

[51] Hofmann PA, Fuchs F. Effect of length and cross-bridge attachment on Ca^2+^ binding to cardiac troponin C. Am J Physiol Cell Physiol 253: C90–C96, 1987. doi:10.1152/AJPCELL.1987.253.1.C90. 2955701

[52] Robertson SP, Johnson JD, Holroyde MJ, Kranias EG, Potter JD, Solaro RJ. The effect of troponin I phosphorylation on the Ca^2+^-binding properties of the Ca^2+^-regulatory site of bovine cardiac troponin. J Biol Chem 257: 260–263, 1982. doi:10.1016/s0021-9258(19)68355-9. 7053370

[53] Spurgeon HA, DuBell WH, Stern MD, Sollott SJ, Ziman BD, Silverman HS, Capogrossi MC, Talo A, Lakatta EG. Cytosolic calcium and myofilaments in single rat cardiac myocytes achieve a dynamic equilibrium during twitch relaxation. J Physiol 447: 83–102, 1992. doi:10.1113/jphysiol.1992.sp018992. 1593465 PMC1176026

[54] Allen DG, Kentish JC. The cellular basis of the length-tension relation in cardiac muscle. J Mol Cell Cardiol 17: 821–840, 1985. doi:10.1016/S0022-2828(85)80097-3. 3900426

[55] Irving TC, Konhilas J, Perry D, Fischetti R, De Tombe PP. Myofilament lattice spacing as a function of sarcomere length in isolated rat myocardium. Am J Physiol Heart Circ Physiol 279: H2568–H2573, 2000. doi:10.1152/ajpheart.2000.279.5.h2568. 11045995

[56] Smith L, Tainter C, Regnier M, Martyn DA. Cooperative cross-bridge activation of thin filaments contributes to the Frank-Starling mechanism in cardiac muscle. Biophys J 96: 3692–3702, 2009. doi:10.1016/j.bpj.2009.02.018. 19413974 PMC3325146

[57] Fabiato A, Fabiato F. Myofilament-generated tension oscillations during partial calcium activation and activation dependence of the sarcomere length-tension relation of skinned cardiac cells. J Gen Physiol 72: 667–699, 1978. doi:10.1085/jgp.72.5.667. 739258 PMC2228558

[58] Farman GP, Allen EJ, Schoenfelt KQ, Backx PH, De Tombe PP. The role of thin filament cooperativity in cardiac length-dependent calcium activation. Biophys J 99: 2978–2986, 2010. doi:10.1016/j.bpj.2010.09.003. 21044595 PMC2965940

[59] Kajiwara H, Morimoto S, Fukuda N, Ohtsuki I, Kurihara S. Effect of troponin I phosphorylation by protein kinase A on length-dependence of tension activation in skinned cardiac muscle fibers. Biochem Biophys Res Commun 272: 104–110, 2000. doi:10.1006/bbrc.2000.2741. 10872811

[60] Soller KJ, Yang J, Veglia G, Bowser MT. Reversal of phospholamban inhibition of the sarco(endo)plasmic reticulum Ca^2+^-ATPase (SERCA) using short, protein-interacting RNAs and oligonucleotide analogs. J Biol Chem 291: 21510–21518, 2016. doi:10.1074/jbc.M116.738807. 27531746 PMC5076822

[61] Kurihara S, Komukai K. Tension-dependent changes of the intracellular Ca^2+^ transients in ferret ventricular muscles. J Physiol 489: 617–625, 1995. doi:10.1113/jphysiol.1995.sp021077. 8788928 PMC1156833

[62] Grynkiewicz G, Poenie M, Tsien RY. A new generation of Ca^2+^ indicators with greatly improved fluorescence properties. J Biol Chem 260: 3440–3450, 1985. 3838314

[63] Highsmith S, Bloebaum P, Snowdowne KW. Sarcoplasmic reticulum interacts with the Ca^2+^ indicator precursor Fura-2-AM. Biochem Biophys Res Commun 138: 1153–1162, 1986. doi:10.1016/S0006-291x(86)80403-x. 3755905

[64] Tsien RY. Fluorescent probes of cell signaling. Annu Rev Neurosci 12: 227–253, 1989. doi:10.1146/annurev.ne.12.030189.001303. 2648950

[65] Lukács GL, Kapus A, Fonyó A. Parallel measurement of oxoglutarate dehydrogenase activity and matrix free Ca2+ in fura-2-loaded heart mitochondria. FEBS Lett 229: 219–223, 1988. doi:10.1016/0014-5793(88)80831-7. 2450043

[66] Malgaroli A, Milani D, Meldolesi J, Pozzan T. Fura-2 measurement of cytosolic free Ca^2+^ in monolayers and suspensions of various types of animal cells. J Cell Biol 105: 2145–2155, 1987. doi:10.1083/jcb.105.5.2145. 3680375 PMC2114834

[67] Poenie M, Alderton J, Steinhardt R, Tsien R. Calcium rises abruptly and briefly throughout the cell at the onset of anaphase. Science 233: 886–889, 1986. doi:10.1126/science.3755550. 3755550

[68] Sipido KR, Wier WG. Flux of Ca^2+^ across the sarcoplasmic reticulum of guinea‐pig cardiac cells during excitation‐contraction coupling. J Physiol 435: 605–630, 1991. doi:10.1113/jphysiol.1991.sp018528. 1770453 PMC1181480

[69] Sparrow AJ, Sievert K, Patel S, Chang Y-F, Broyles CN, Brook FA, Watkins H, Geeves MA, Redwood CS, Robinson P, Daniels MJ. Measurement of myofilament-localized calcium dynamics in adult cardiomyocytes and the effect of hypertrophic cardiomyopathy mutations. Circ Res 124: 1228–1239, 2019. doi:10.1161/CIRCRESAHA.118.314600. 30732532 PMC6485313

[70] Vetter AD, Martin AA, Thompson BR, Thomas DD, Metzger JM. Sarcomere integrated biosensor detects myofilament-activating ligands in real time during twitch contractions in live cardiac muscle. J Mol Cell Cardiol 147: 49–61, 2020. doi:10.1016/j.yjmcc.2020.07.012. 32791214 PMC7584764

[71] Grimm AF, Lin HL, Grimm BR. Left ventricular free wall and intraventricular pressure-sarcomere length distributions. Am J Physiol Heart Circ Physiol 8: H101–H107, 1980. doi:10.1152/ajpheart.1980.239.1.h101. 7396007

[72] Pollack GH, Huntsman LL. Sarcomere length–active force relations in living mammalian cardiac muscle. Am J Physiol 227: 383–389, 1974. doi:10.1152/ajplegacy.1974.227.2.383. 4854194

[73] Rodriguez EK, Hunter WC, Royce MJ, Leppo MK, Douglas AS, Weisman HF. A method to reconstruct myocardial sarcomere lengths and orientations at transmural sites in beating canine hearts. Am J Physiol Heart Circ Physiol 263: H293–H306, 1992. doi:10.1152/ajpheart.1992.263.1.h293. 1636767

[74] Sonnenblick EH, Ross J Jr, Covell JW, Spotnitz HM, Spiro D. The ultrastructure of the heart in systole and diastole. Chantes in sarcomere length. Circ Res 21: 423–431, 1967. doi:10.1161/01.RES.21.4.423. 6057700

[75] Janssen PML, De Tombe PP. Uncontrolled sarcomere shortening increases intracellular Ca^2+^ transient in rat cardiac trabeculae. Am J Physiol Heart Circ Physiol 272: H1892–H1897, 1997. doi:10.1152/ajpheart.1997.272.4.h1892. 9139976

